# Medicinal plants from swidden fallows and sacred forest of the Karen and the Lawa in Thailand

**DOI:** 10.1186/1746-4269-9-44

**Published:** 2013-06-24

**Authors:** Auemporn Junsongduang, Henrik Balslev, Angkhana Inta, Arunothai Jampeetong, Prasit Wangpakapattanawong

**Affiliations:** 1 Department of Biology, Faculty of Science, Chiang Mai University, Huaykaew Road, Chiang Mai 50200, Thailand; 2 Department of Bioscience, Ecoinformatics and Biodiversity Group, Aarhus University, Building 1540, Ny Munkegade 114-116, DK-8000, Aarhus C, Denmark

**Keywords:** Ethnomedicinal plants, Protected forests, Traditional knowledge, Tribal community

## Abstract

**Background:**

Many ecosystem services provided by forests are important for the livelihoods of indigenous people. Sacred forests are used for traditional practices by the ethnic minorities in northern Thailand and they protect these forests that are important for their culture and daily life. Swidden fallow fields are a dominant feature of the agricultural farming landscapes in the region. In this study we evaluate and compare the importance of swidden fallow fields and sacred forests as providers of medicinal plants among the Karen and Lawa ethnic minorities in northern Thailand.

**Methods:**

We made plant inventories in swidden fallow fields of three different ages (1–2, 3–4, 5–6 years old) and in sacred forests around two villages using a replicated stratified design of vegetation plots. Subsequently we interviewed the villagers, using semi-structured questionnaires, to assess the medicinal use of the species encountered in the vegetation survey.

**Results:**

We registered a total of 365 species in 244 genera and 82 families. Of these 72(19%) species in 60(24%) genera and 32(39%) families had medicinal uses. Although the sacred forest overall housed more species than the swidden fallow fields, about equal numbers of medicinal plants were derived from the forest and the fallows. This in turn means that a higher proportion (48% and 34%) of the species in the relatively species poor fallows were used for medicinal purposes than the proportion of medicinal plants from the sacred forest which accounted for 17–22%. Of the 32 medicinal plant families Euphorbiaceae and Lauraceae had most used species in the Karen and Lawa villages respectively.

**Conclusion:**

Sacred forest are important for providing medicinal plant species to the Karen and Lawa communities in northern Thailand, but the swidden fallows around the villages are equally important in terms of absolute numbers of medicinal plant species, and more important if counted as proportion of the total number of species in a habitat. This points to the importance of secondary vegetation as provider of medicinal plants around rural villages as seen elsewhere in the tropics.

## Background

Ecosystem services and goods have received much attention in recent years. Typically services and goods include 1) supply of valuable commodities and materials such as agricultural-, forest-, mineral-, and pharmaceutical products, 2) support and regulation of environmental conditions through flood control, water purification, pollination, and a number of other similar processes and 3) provision of cultural and aesthetic benefits that may also be the basis for ecotourism [1].

As in many tropical regions, shifting cultivation is a major land use system in northern Thailand and it is a major driver of deforestation in the upland areas [2,3]. About 5% of the original forested areas in northern Thailand are under shifting cultivation [3] and fallow forests cover large parts of the highlands of this region [4]. Under this land use system fields are abandoned after cultivation and are left without intensive use for 5–15 years to regenerate to forest before they are again turned into crop cultivation [5]. The impact of this ecological transformation on the availability of usable plants is not well understood, and there is little research concerning the habitats from which shifting cultivators gather wild plants. Nevertheless the shifting cultivators still obtain many of the plants that they need for their livelihood from these fallow fields and regenerating forests [6].

Sacred forests are segments of the landscape that represents old traditions of preserving climax forest patches based on local culture and religious beliefs and they are found throughout the world. A sacred forest represents a functional link between cultural life and the forest management system of a region. Sacred forests have been studied in many parts of the world including Africa, [7], China [8], and especially in India [9-13]. Ethnobotanical studies of sacred forest in India [14-16] have documented informal management systems of sacred forest that not only conserve useful species, but also harbor many unique plants for which local people have discovered medicinal values [17,18]. Sacred forests are often seen as reservoirs of local biodiversity that preserve a unique fauna and flora including their medicinal plants [11]. Depending on location and management, sacred forest provide a number of other ecosystem services such as cultural amenities but many of these aspects remain poorly explored [19]. Ecosystem services in the form of medicinal plants from sacred forest can be important for indigenous people in remote areas, since many rural communities depend on wild plants for their diet and livelihood [20]. In Thailand there are many different types and sizes of sacred forests, ranging from a single tree to large forests that sometimes cover entire mountains [21]. In northern Thailand sacred forests are geographically dispersed and often associated with ethnic minorities living in the mountains [22]. Local laws and customs usually limit the villagers’ activities in these forests. Hunting, grazing, and logging may be prohibited or restricted and villagers are consciencious not to damage them [23]. The ethnobotany of sacred forests has never been studied in Thailand.

Simplistic views of ethnoecological relationships between ethnic groups and their surrounding ecosystems often view the untouched virgin species rich forests as the main provider of useful plants, whereas secondary vegetation is often seen as degraded and useless. A growing body of evidence however points to these secondary recovering ecosystems as important providers of useful plants. Examples of how secondary vegetation make important contributions to the provision of useful plants come from the Amazon and the Atlantic forests in South America [24-26] and from Vietnam [27]. Here we study this phenomenon, which appears to be general, and we test whether it also occurs in a fallow/sacred-forest cultural landscape mosaic in northern Thailand. The objectives of our study were to examine the ecosystem services from swidden fallow fields of different ages and adjacent sacred forests and in particular to compare how these different habitats provide medicinal plants in two ethnic minority communities in northern Thailand, one of the Karen and one of the Lawa. Specifically we asked the following questions: 1) How species rich are the fallow fields of different ages and the sacred forests and how many of their species have medicinal uses? 2) Of the species encountered how many are derived from each habitat and how many medicinal plants are provided by each habitat?

## Materials and methods

### Study areas

The study area is in Mae Cheam watershed in northern Thailand approximately 75 km southwest of the city of Chiang Mai. This watershed is important for its biodiversity and its varied forest types and vegetation and in addition it is inhabited by several ethnic minority groups [28]. Our study was focused on two villages of different ethnic groups, the Karen village Mae Hae Tai and the Lawa village Mude Lhong (Table 1). The Karen is the largest of ethnic group in Thailand [29]. There are four groups of Karens; the Sgaw Karen, the B’ghwe Karen, the Pa-O Karen or Thaung thu and the Pwo Karen [30]. Karen-Sgaw is the largest group in Thailand and also in the Mae Cheam watershed- [29]. The Karen are autonomous and economically self-sufficient and live in remote and isolated areas and have rituals that focus on living in harmony with the nature that surrounds them [31,32]. The Lawa do not live outside of Thailand and are sometimes not counted among the hill tribes. The history of the Lawa is long and poorly understood [31]. Regardless of such disagreements about their assignment, the Lawa are a minority group in the northern Thailand [29]. Their economy is based on agriculture, with rice grown according to a sophisticated rotation shifting cultivation system [33]. The two villages are surrounded by several different habitats such as sacred forests, rice fields and swidden fallow fields of different ages. Villagers are only allowed, by the village committee, to extract minor forest products from the sacred forests in quantities that must be agreed upon [33]. In Mae Hae Tai (Karen), which is mainly Christian, villagers maintain traditional beliefs related to the forest that surrounds them and they worship the forest in tree ordination ceremonies to raise awareness of environmental protection and to build a spiritual commitment to conserve the forests and the watersheds [23]. In Mude Lhong (Lawa) which is Animistic-Buddhist [34] the inhabitants practice extensive traditional customs through animists beliefs related to protecting their environment, rivers and forests. The sacred forest in Mae Cheam occur in a matrix of cultivated fields and fallows, which, in this watershed, are up to six years old although fallows may be up to 15 years old elsewhere.

**Table 1:** Baseline information for the two villages, a Karen and a Lawa village, in northern Thailand where medicinal plants were studied

**Village**	**Mae Hae Tai**	**Mude Lhong**
Ethnicity	Karen	Lawa
Religion	Christian	Animists-Buddhism
Co-ordinates	18°25′37.0″ N, 98°8′12.7″ E	18° 28′ 0.5″ N , 98° 11′ 25.5″ E
Elevation (m) a.s.l.	1,090	950
Households	67	55
Population (males/females)	346 (172/173)	286 (136/150)
Distance to nearest town (km)	53	48
Total size of sacred forest (acres/ha)	804/325	815/330
Total size of swiden fallow fields (acres/ha)	1,043/422	1,457/590
Permanent cash crops	Cabbage (*Brassica oleracea* L.), Coffee (*Coffea arabica* L.)	Cabbage *(Brassica oleracea* L.), Onion (*Allium ascalonicum* L.), Flint corn (*Zey mays* L.)

### Data sampling

We established sampling plots around both villages in 2009 and 2010 in the sacred forest and swidden fallow fields of various ages (young fallow, 1–2 years; medium fallow, 3–4 years; old fallow, 5–6 years). Three plots (20 × 40 m) were laid out parallel to contour lines and these three plots were replicated in each habitat. In the 24 plots (total 1.92 ha) all plant species were collected and later identified at the Queen Sirikit Botanic Garden Herbarium (QSBG) with the help of taxonomic specialists J. F. Maxwell and M. Norsaengsri. Voucher specimens are deposited at the herbaria of the Department of Biology, Chiang Mai University and at Queen Sirikit Botanic Garden Herbarium (QSBG), Chiang Mai, Thailand. Based on species lists derived from the vegetation surveys of each habitat type, ethnobotanical data were gathered between August, 2011 and February, 2012 using semi-structured interviews. Our informants were villagers who were born and had always lived in the communities and their ages ranged from 15–84 years. Photographs of plants and freshly collected material from the swidden fallow fields and sacred forest were shown to the informants following established interview techniques [35,36]. The interviews were done in Karen and Lawa with the help of an interpreter. We made 35 interviews in the Karen village and 32 in the Lawa village corresponding to 10% and 11% their populations (Table 1). Prior to the start of interviews concerning the medicinal use of plants in the Karen village Mae Hat Tai and the Lawa village Mude Lhong communal meetings were held with all inhabitants, including the village leaders, during which the purpose and the methods of the study was explained and approved. It was agreed that the obtained results would be shared with the villagers in the form of a popular publication once the research had been formally published. In addition it was agreed that all informants would be asked for their prior informed consent individually before any interview was undertaken. Consequently such consent was obtained for each interview performed.

### Data analyses

Jaccard’s Index (JI) was used to determine the similarity of medicinal plants species [37], which is based on the presence or absence of species on each list. Relating the number of species in common to the total number, it is expressed as:

$$\mathrm{JI}=\left(\frac{c}{a+b+c}\right)\times 100$$

Where *a* is the number of species unique to area A and *b* is the number of species unique to area B, and *c* is the number of species found in both areas.

Use Value was calculated to determine the most important medicinal plant species in each habitat [38],

$$\mathrm{UV}=\frac{\mathrm{Ui}}{N}$$

Where *Ui* is the number of use-reports cited by each informant for a given species in each habitat and *N* is the total number of informants.

Linear regression was done to account for correlated responses between the age of fallow fields and total number of medicinal plants in each sampling sites. Chi-square test was used to analyze differences between habitat and number of medicinal plants species in the two villages and to analyze if the sources of medicinal plants depend on the habitat. All analyses were done with the SPSS 16.0 software package for Windows.

## Results and discussion

### Species richness, number of medicinal plants and their taxonomic diversity

**Figure 1:**
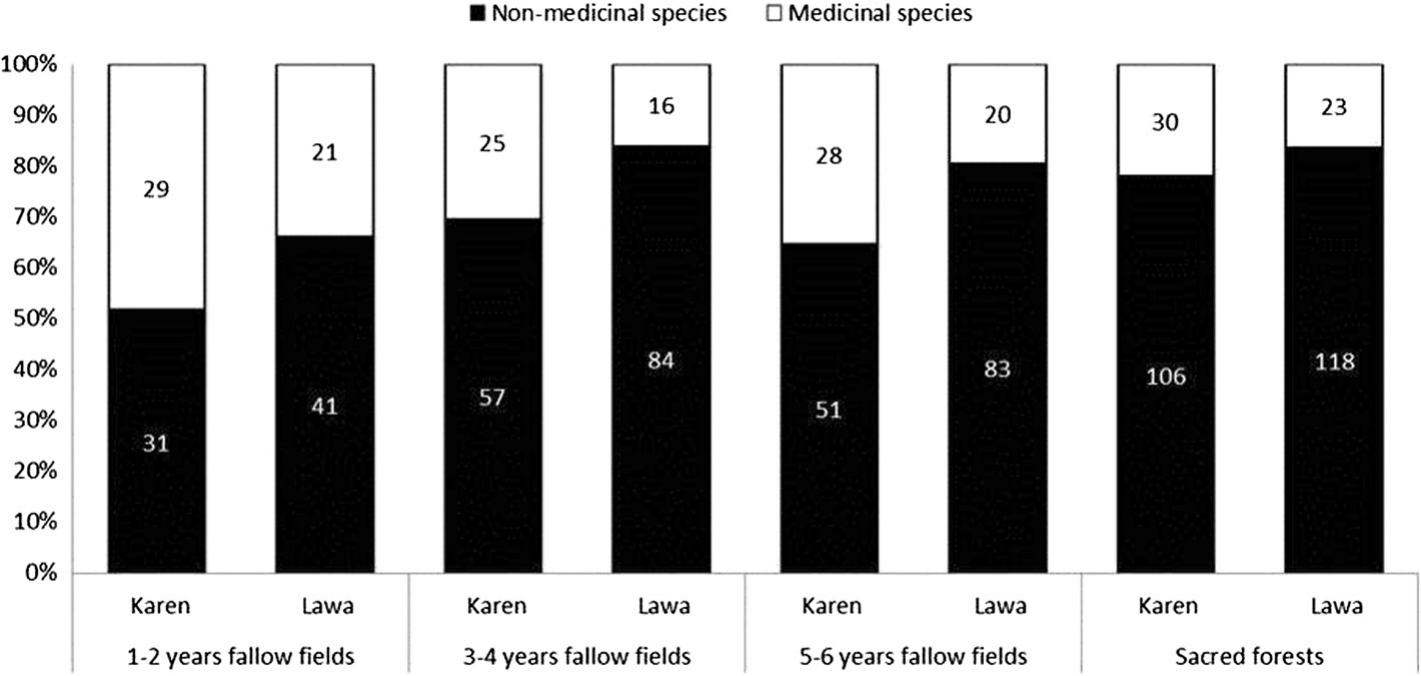
Total number and proportions of medicinal plants and non-medicinal plants in four different habitats surrounding a Karen and a Lawa village in the Mae Cheam watershed in northern Thailand.

**Figure 2:**
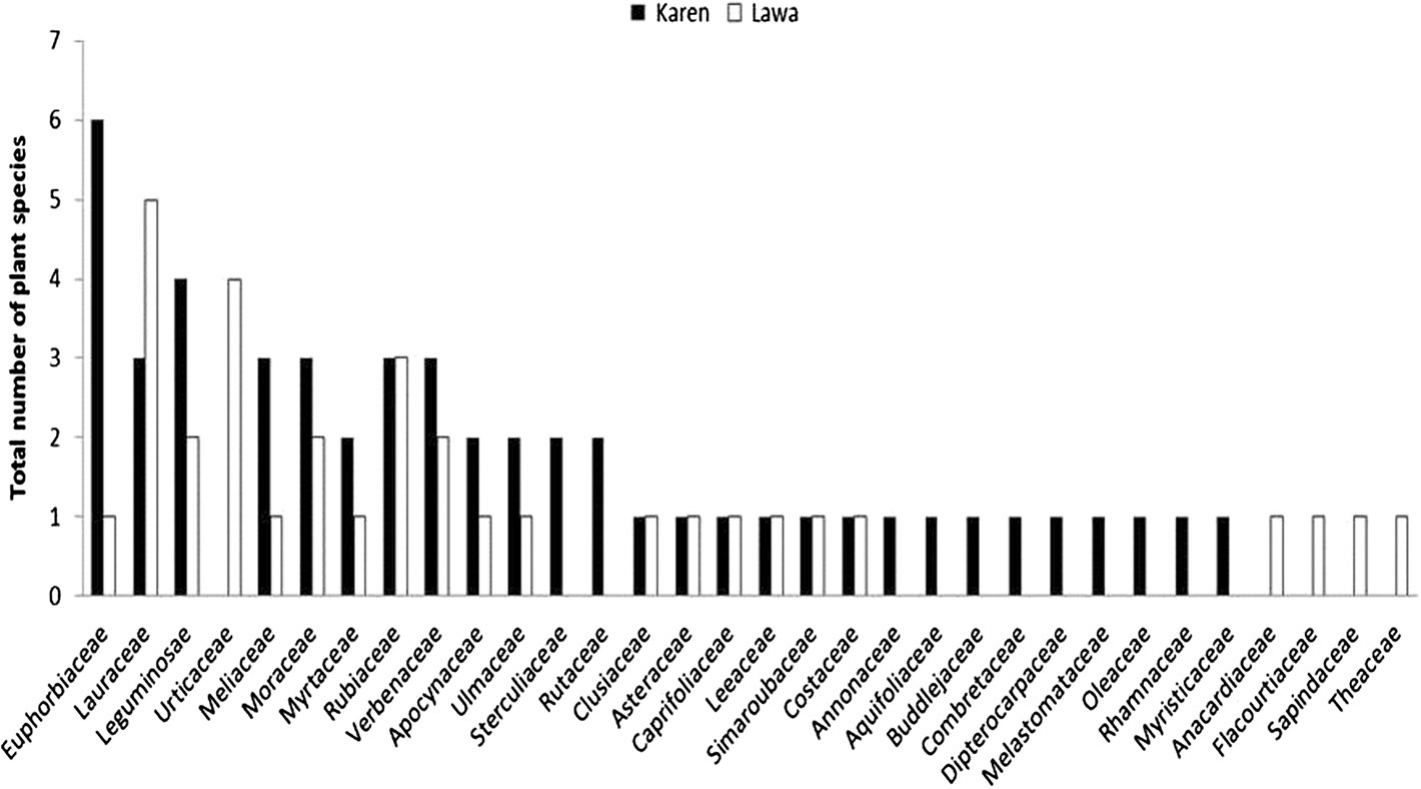
Number of medicinal species per plant family in a Karen and a Lawa village in the Mae Cheam watershed in northern Thailand.

In total we registered 365 species, 245 in the Karen village and 240 in the Lawa village. The highest species richness was found in the sacred forests of both villages and the lowest number of species was found in the youngest (1–2 years old) fallow fields (Figure 1). We encountered 72 different species of medicinal plants belonging to 32 families and 60 genera (Table 2). Of these, 50 species in 44 genera and 27 families were used by the Karen and 32 species in 30 genera and 21 families were used by the Lawa. The most used plant families were Euphorbiaceae (6 species) in the Karen village and Lauraceae (5 species) in the Lawa village (Figure 2). Eleven families were used only by the Karen and not by the Lawa whereas five families were used only by the Lawa and not by the Karen (Figure 2). Only 15% of the medicinal plants (11 species) were shared among the two villages (Table 2). Most of the plant families that were used exclusively in one of the villages were represented by a single species, but it is noteworthy that Urticaceae had four medicinal species in the Lawa village and none in the Karen village.

**Table 2:** List of medicinal plants used by the villagers in a Karen and a Lawa village in the Mae Cheam watershed in northern Thailand

** Species – Family (Voucher no.)**	** Local name**		**Source habitat of Medicinal plants**							
			** Swidden cultivation fields**						** Sacred forest**	
			** 1-2 Years**		** 3-4 Years**		** 5-6 Years**			
	**Karen**	**Lawa**	**Karen**	**Lawa**	**Karen**	**Lawa**	**Karen**	**Lawa**	**Karen**	**Lawa**
*Acacia concinna* (Willd.) DC- LEGU (AJK004, AJL001)	Po chi sa	Som poi	-	√	-	-	√	-	-	√
*Acrocarpus fraxinifolius* Wight ex Arn. – LEGU (AJK 048)	Law bor dey	-	√	-	√		√	-	-	-
*Actinodaphine henryi* Gamb. – LAUR (AJL 164)	-	Coh yeum ngo	-	-	-	-	-	-	-	√
*Aglaia elliptica* Blume – MELI (AJK 141, AJL 136)	Sey leu sa, Sey ney sa, Tur see sor	Gor dong pia	√	√	√	√	√	√	√	√
*Aglaia lawii* (Wight) Sald. ex Rama. – MELI (AJK 200)	Sey pi	-	-	-	-	-	-	-	√	-
*Alstonia scholaris* (L.) R. Br. – APOC (AJK 003, AJL 063)	Nor bey, Pa bor eu	Hyar, Sa weing	√	-	√	-	√	√	-	√
*Aphananthe aspera* (Thunb.) Planch. - ULMA (AJK 230)	Pore loo too, Sa deui cwa	-	√	-	-	-	-	-	-	-
*Artocarpus nitidus* Trec.*-* MORA (AJK 246)	Pa da soi	-	-	-	-	-	-	-	√	-
*Bauhinia glauca* (Wall. ex Bth.) Bth. ssp. *tenuiflora* (Watt ex Cl.) K. & S.S. Lar.*-* LEGU (AJK 245)	Per na meu too	-	-	-	-	-	-	-	√	-
*Bochmeria nivea* (L.) Gaud. var. *tenacissima* (Roxb.) Miq. -URTI (AJL 090)	-	Hyew	-	√	-	**-**	-	-	-	√
*Boehmeria malabarica* Wall. ex Wedd. –URTI (AJL 040)	-	Kang poi	-	√	-	√	-	√	-	√
*Breynia retusa* (Dennst.) Alst.- EUPH (AJK 179)	Mi ni mey	-	-	-	-	-	-	-	√	-
*Brucea mollis* Wall.- SIMA (AJK 045)	Sey gor wey	-	-	-	√	-	√	-	√	-
*Buddleja asiatica* Lour.- BUDD (AJK 034)	Pore gi braa	-	√	-	√	-	√	-	√	-
*Callicarpa arborea* Roxb. var. *arborea* – VERB (AJK 023)	Poh qui	-	√	-	√	-	√	-	√	-
*Calophyllum polyanthum* Wall. ex Choisy- CLUS (AJK 022)	Seu mee la	-	√	-	√	-	√	-	-	-
*Catunaregam spathulifolia* Tirveng.*-* RUBI (AJK 204)	Puoi sa mu	-	√	-	√	-	√	-	-	-
*Celtis tetrandra* Roxb.- ULMA (AJL 072)	-	Coh tar, Ha tong soo	-	√	-	-	-	-	-	-
*Chionanthus ramiflorus* Roxb.- OLEA (AJK 148)	Bey plor sa	-	-	-	√	-	-	-	-	-
*Chisocheton cumingianus* (C. DC.) Harms ssp. *balansae* (C.DC.) Mabb.- MELI (AJK 100)	Sa me jeu	-	-	-	-	-	-	-	√	-
*Chromolaena odorata* (L.) R. M. King & H. Rob.*-* ASTE (AJK 066, AJL 220)	Chor per gwe	Piaw sa non chime	√	√	√	-	√	-	-	-
*Cinnamomum iners* Reinw. ex Bl. –LAUR (AJL 044)	-	Bai herng, My hoam	-	√	-	√	-	√	-	√
*Clerodendrum serratum* (L.) Moon var. *serratum*- VERB (AJL 120)	-	Coh song sam	-	√	-	√	-	√	-	√
*Colebrookia oppositifolia* Smith- VERB (AJL 155)	-	Coh tia gleing	-	√	-	√	-	√	-	-
*Costus speciosus* (Koeh.) J.E. Sm. var. *speciosus* – COST (AJK 002, AJL 088)	Su ley bo	Gu gi, Toh toi	√	√	√	√	√	√	-	√
*Cratoxylum formosum* (Jack.) Dyer ssp. *pruniflorum* (Kurz) Gog.- CLUS (AJL 097)	-	Gu gi, Toh toi, Sa nung kai	-	√	-	√	-	√	-	√
*Dalbergia cultrata* Grah. ex Bth.- LEGU (AJL 217)	-	Hyu	-	-	-	√	-	√	-	-
*Dendrocnide stimulans* (L.f.) Chew- URTI (AJL 147)	-	Tug kleing, Dian	-	√	-	√	-	√	-	-
*Desmos dumosus* (Roxb.) Saff. var. *glabrior* Craib *-* ANNO (AJK 120)	Pore na seu	-	-	-	-	-	-	-	√	-
*Eugenia cumini* (L.) Druce var. *cumini* – MYRT (AJK 032)	Sey mee su, Sey grey gwa	-	√	-	-	-	√	-	-	-
*Eugenia fruticosa* (Roxb. ex DC.) Roxb. –MYRT (AJK 074)	Sir me	-	√	-	√	-	√	-	√	-
*Eurya accuminata* DC.- THEA (AJL 029)	-	Coh joung, Coh hmoi	-	√	-	√	-	√	-	-
*Ficus auriculata* Lour. – MORA (AJK 007)	Ta geu ha	-	-	-	-	-	√	-	√	-
*Ficus carpillipes* Gagnep.- MORA (AJL 087)	-	Ye ya gor	-	-	-	-	-	-	√	-
*Ficus virens* Aiton var. *virens*- MORA (AJK 082)	Clur sa	-	-	-	-	-	-	-	√	-
*Flacourtia indica* (Blume) Merr.*-* FLAC (AJL 234)	-	Mi gai	-	-	-	-	-	-	-	√
*Glochidion eriocarpum* Champ. – EUPH (AJK 065)	Sey pore meu pra	-	√	-	-	-	-	-	√	-
*Glochidion sphaerogynum* (M.A.) Kurz *–* EUPH (AJK 067)	Tur si phlaa	-	√	-	√	-	√	-	√	-
*Gmelina arborea* Roxb*.* –VERB (AJK 252, AJL 083)	Sey gor wey	Ga hor	√	-	√	-	√	√	√	√
*Helicteaes hirsuta* Lour.- STER (AJK 181)	Poa ji gwey	-	√	-	√	-	√	-	√	-
*Helicteres elongata* Wall. ex Boj. *–* STER (AJK 121)	Ta gor eh	-	√	-	√	-	√	-	-	-
*Horsfieldia amygdalina* (Wall.) Warb. var. *amygdalina* – MYRI (AJK 129, AJL 137)	Poo see sho	Pley coh	-	-	-	-	√	√	-	√
*Ilex umbellulata* (Wall.) Loes.*-* AQUI (AJK 199)	Bley bor sa	-	√	-	-	-	-	-	√	-
*Indigofera tinctoria* Linn. – LEGU (AJK 244)	Sor me moo boa coa	-	-	-	-	-	-	-	√	-
*Kopsia aborea Blume -* APOC (AJK 182)	Ti chi cho por	-	-	-	-	-	-	-	√	-
*Leea indica* (Burm. F.) Merr. - LEEA (AJK 131, AJL 080)	Sey bor sa	Dird	√	√	√	√	√	√	-	√
*Litsea cubeba* (Lour.) Pers. var. *cubeba* –LAUR (AJL 008)	-	Coh loh	-	√	-	√	-	√	-	√
*Litsea elongata* (Wall. ex Nees) Bth. & Hk.f. – LAUR (AJK 127)	Nor tu leu	-	√	-	-	-	-	-	√	-
*Litsia monopetala* (Roxb.) Pers.- LAUR (AJK 154, AJL 165)	Pey jeu ya	Hyum ngo, Hyeung	√	-	-	√	√	√	-	√
*Mallotus sp. –* EUPH (AJL 235)	-	Co wan	-	√	-	√	-	√	-	√
*Mangifera coloneura Kurz –*ANAC (AJL 233)	-	Coh pae	-	-	-	-	-	√	-	-
*Maoutia puva* (Wall. ex Hook.) Wedd.-URTI (AJL 177)	-	Hyei	-	√	-	-	-	√	-	√
*Melastoma malabathricum* L. ssp. *norman* D. Don K. Meyer*-* MELA (AJK 019)	Sey la pley	-	√	-	√	-	√	-	-	-
*Melicope pteleifolia* (Champ. ex Bth.) T. Hari- RUTA (AJK 250)	Pa sa ley	-	√	-	√	-	√	-	-	-
*Millettia pachycarpa* Bth.- LEGU (AJK 084)	Cher dui meu	-	√	-	√	-	-	-	√	-
*Mussaenda parva* Wall. ex. G. Don - RUBI (AJK 191)	Go wa sa	-	√	-	√	-	√	-	-	-
*Pavetta indica* L.- RUBI (AJL 113)	-	Coh ca tok	-	√	-	-		-	-	-
*Phoebe lanceolata* (Nees) Nees –LAUR (AJK 047, AJL 122)	Sey glow bow	Coh sa loh, Hyom hngo	√	√	√	√	√	√	√	√
*Phyllanthus emblica* L.-EUPH (AJK 090)	Sey ya sa		-	-	-	-	-	-	√	-
*Picrasma javanica* Bl.- SIMA (AJL 182)		Sa geun	-	-	√	-	-	-	-	√
*Sambucus javanica* Reinw. ex Blume-CAPR (AJK 088, AJL 026)	Ta si ga jeu	La oil toui	√	√	√	√	√	√	√	-
*Sapindus rarak* DC. – SAPI (AJL 025)	-	Glerw	-	-	-	-	-	-	-	√
*Sauropus quadrangularis* (Willd.) M.-A.- EUPH (AJK 144)	Ta chor dor	-	√	-	-	-	-	-	-	-
*Shorea roxburghii* G.Don*-* DIPT (AJK 196)	Sey bey tour, Ta glor	-	-	-	-	-	-	-	√	-
*Tarennoidea wallichi* (Hk.f.) Tirv. &Sastre - RUBI (AJK 113, AJL 093)	Jor tur goh pore	Gud song mum	-	√	-	√	-	√	√	√
*Terminalia chebula* Retz. var. *chebula -* COMB (AJK 057)	Hor chi dor, Por hor sa	Ga gai, Bur	√	-	√	-	√	-	-	-
*Toddalia asiatica* (L.) Lmk. – RUTA (AJK 005)	Ta sai iw si, Pca sey ley	-	-	√	-	√	-	√	-	-
*Trema orientalis* (L.) Bl.- ULMA (AJK 076)	Per dor, Sa ley	-	-	-	-	-	-	-	√	-
*Triadica cochinchinensis* Lour- EUPH (AJK 111)	Nor	-	-	-	-	-	-	-	√	-
*Vitex sp.-* VERB (AJK 109)	Tor gloa soo, Seu ca poh jor	-	-	-	-	-	√	-	-	-
*Wendlandia scabra* Kurz. var*. scabra –* RUBI (AJL 051)	-	Coh yong	-	-	-	√	-	-	-	-
*Ziziphus oenoplia* var.*brunoniana* Tardieu Mill- RHAM (AJK 015)	Bla kho dey	-	√	-	√	-	√	-	-	-

The family name of each plant species is indicated by the first four letters in upper case of the Latin family name. Vouchers were collected in the number series of Auemporn Junsongduang (AJK for Karen, and AJL for Lawa) and deposited in the herbaria of the Ethnobotanical Research Unit, Department of Biology, Faculty of Science, Chiang Mai University and Queen Sirikit Botanic Garden Herbarium, Chiang Mai Thailand: √ = present; - = Absent.

*Costus speciosus* var. *speciosus* was the most important medicinal species in the 1–2 years old fallow fields and it had the highest UV in both villages (Table 3). In the Karen village, *Melastoma malabathricum* L. ssp. *norman* and *Eugenia cumini* var. *cumini* were the most important species in 3–4 and 5–6 years old fallow fields, respectively. In the Lawa village, *Phoebe lanceolata* was the most important medicinal plants in 3–4 years old fallow fields and also had highest UV in 5–6 years old fallow fields. In the sacred forest of the Karen *Ficus auriculata* had the highest UV and *Cinnamomum iners* had the highest UV in the sacred forest of the Lawa.

**Table 3:** The most used species of medicinal plants and their UV (Use-Value) in four different habitats around a Karen and a Lawa village in northern Thailand

**Village**	**Habitats**							
	**Fallow fields**						**Sacred forests**	
	**1-2 Years**		**3-4 Years**		**5-6 Years**		**Species**	**UV**
	**Species**	**UV**	**Species**	**UV**	**Species**	**UV**		
Karen	*Costus speciosus* (Koeh.) J.E. Sm. var. *speciosus*	0.42	*Melastoma malabathricum* L. ssp. *norman* D. Don K. Meyer	0.34	*Eugenia cumini* (L.) Druce var. *cumini*	0.34	*Ficus auriculata* Lour.	0.08
Lawa	*Costus speciosus* (Koeh.) J.E. Sm. var. *speciosus*	0.31	*Phoebe lanceolata* (Nees) Nees	0.34	*Phoebe lanceolata* (Nees) Nees	0.37	*Cinnamomum iners* Reinw. ex Bl.	0.12

When compared to sacred forests and swidden fallow fields documented elsewhere in South East Asia and in Africa (Table 4), the species richness and the number of medicinal plants reported here are within the range reported in those other studies.

**Table 4:** Species richness and number of medicinal plant species in sacred forests and swidden falow fields in 11 selected localities in South East Asia and Africa compared to the numbers reported in this study

**Locality**	**Species*richness***		**Medicinal plants (%)**	**Most important families**
**Sacred forest**	**Total**	**Medicinal**		
India, Kodagu district, Karnataka state^1^	241	136	56%	-
India, Meghalaya state ^2^	-	80	-	Lauraceae, Euphorbiaceae
India, Manipur state ^3^	-	120	-	Asteraceae, Verbenaceae
India, Kanyakumari district, Tami Nadu state ^4^	329	34	10%	Rutaceae, Euphorbiaceae
India, Cuddalore district, Taminadu ^5^	-	33	-	Leguminosae, Agavaceae
India, Sikkim state^6^	241	41	17%	-
India, Virudhunagar district, Tamil Nadu state ^7^	-	53	-	Leguminosae, Moraceae
India, Andhra Pradesh state ^8^	-	18	-	Loganiaceae, Leguminosae
Thailand, Mae Cheam district, Chiang Mai province^9^	221	50	22%	Euphorbiaceae, Lauraceae
**Swidden fallow fields**				
Thailand, Mae La Noi district, and Muang district, Mae Hong Son province ^10^	489	84	17%	Euphorbiaceae, Leguminosae
Lao, Pha Oudom district, Bo Kaeo province ^11^	141	58	41%	Leguminosae, Euphorbiaceae
Nigeria, Lagos state ^12^	104	48	46%	Euphorbiaceae, Leguminosae
Thailand, Mae Tang district Chaing Mai province ^13^	295	119	40%	-
Thailand, Mae Cheam district, Chiang Mai province ^14^	218	75	34%	Euphorbiaceae, Lauraceae

^1^[14], ^2^[15], ^3^[16], ^4^[17], ^5^[18], ^6^[39],^7^[40], ^8^[41], ^9^ This study, ^10^[4], ^11^[6], ^12^[42], ^13^[43], ^14^ This study.

Euphorbiaceae, which was the most important medicinal plant family in the Karen village, is common among medicinal plant families from sacred forests and swidden fallow fields elsewhere. The Lauraceae, which was the dominant medicinal plant family in the Lawa village, is important in only one of the other studies cited, *i.e.,* from Megalhay in India (Table 4). The two villages studied here are quite different in terms of the taxonomic origin of the medicinal plants (Figure 2) demonstrating that these two cultures, even if living in a shared habitat, have developed taxonomically different medicinal plant systems.

### Sources of the medicinal plants

The overall species richness increased from young over old fallow fields to sacred forest, and about equal numbers of medicinal plant species were derived from the four different habitat types around the villages (Figure 1). Overall the number of medicinal plant species from each habitat varied from 16–30. The differences were not significant, neither overall (χ^2^ = 1.62, *df* = 3, *p* = 0.65) nor when the villages were tested separately (Karen: χ^2^ = 0.50, *df* = 3, *p* = 0.91; Lawa: χ^2^ = 1.30, *df* = 3, *p* = 0.72). Linear regression test in both villages showed that the age of the fallow fields was a weak factor and had negatively significant effect on the total number of medicinal plants (R^2^ = 0.014, Coefficients = −1.181, F = 6.224, *p* = 0.01) and also negative effect in each village but without significant differences (Karen; R^2^ = 0.044, Coefficients = −0.513, F = 3.606, *p* = 0.06: Lawa; R^2^ = 0.044, Coefficients = −0.523, F = 3.606, *p* = 0.06). This explains that the age of fallow did not affect the total number of medicinal plants. So although the sacred forest is much older and richer in species than the fallow fields, they do not provide higher number of medicinal plant species (Figure 1).

Because the four habitat types provide roughly similar numbers of medicinal plants even if their overall species richness is significantly different, the proportion of the species that is used medicinally of a given habitat is greatly different. The young (1–2 years) fallow fields have few species but 48% and 34% of them are used medicinally by the Karen and the Lawa, respectively. In the species rich sacred forests, in contrast, only 22% and 17% of the species are used medicinally (Figure 1). The overall proportion of medicinal plants and non-medicinal plants in each habitat in the two villages were significantly different (χ^2^ = 19.30, *df* = 3, *p* = 0.00) also when the village were tested separately (Karen: χ^2^ = 10.57, *df* = 3, *p* = 0.01; Lawa: χ^2^ = 21.00, *df* = 3, *p* = 0.00).

The swidden fallow fields of different ages of regeneration and the sacred forests provided about equal numbers of medicinal plant species to the two villages. This is surprising when seen in the light of the much higher overall species richness of the sacred forest compared to the surrounding swidden fallow fields. The more intense use of the secondary vegetation of the fallows may be because they are closer to where the villagers have their houses. Another possible explanation may be discouragement coming from the village council’s desire to conserve the sacred forest. The fallow fields, in contrast, are part of the productive land surrounding the villages and the swidden fallows belong to individual villagers which eliminates any problem related to ownership, *etc.* It is interesting that the most recently abandoned field, *i.e.,* the swidden fallows that are 1–2 years old, have the highest proportion of their species being used medicinally. This preference for using secondary vegetation as a source of medicinal plants has previously been demonstrated in the Atlantic Forest of Brazil [24] and also among the ribeirinhos of Amazonian Brazil [26], in dry forest of northeastern Brazil [25] and in Vietnam [27]. It appears that different forests are used and valued differentially, not only with regard to usefulness but also in symbolic-religious terms and together they protect traditional botanical knowledge, people’s health and forests. Nonetheless, sacred forests remain important as providers of medicinal resources in the tribal communities not only in Thailand but also elsewhere in the region.

## Conclusion

Sacred forest and their surrounding fallow fields of different age of regeneration provided approximately the same number of medicinal plant species to both villages. Because the fallow fields were less species rich, the proportion of their species with medicinal uses was consequently higher. Sacred forests are conserved as community forest and they make up a network of protected forest in northern Thailand [22]. Nevertheless it seems, as we document here, that fallow fields after swidden cultivation are equally important as providers of medicinal plants to the ethnic minorities in northern Thailand.

## Competing interests

The authors declare that they have no competing interests.

## Authors’ contributions

The article was initiated by AJ, who recorded and analysis data and prepared the first write-up of the manuscript. HB has critically edited and shaped subsequent versions. AI, AJ, PW have read and approved the final version of the manuscript. All authors read and approved the final manuscript.

## Authors’ information

AJ is a PhD student at the University of Chiang Mai, Thailand, under supervision of associate professor PW and co-supervision of AI and AJ, assistant professors at University of Chiang Mai and members of the Ethnobotany research group. HB is professor at Aarhus University, Bioscience, and functions as external supervisor to AJ and as host to her long term visit to Aarhus University.
